# The Flash-Lag Effect as a Motion-Based Predictive Shift

**DOI:** 10.1371/journal.pcbi.1005068

**Published:** 2017-01-26

**Authors:** Mina A. Khoei, Guillaume S. Masson, Laurent U. Perrinet

**Affiliations:** Institut de Neurosciences de la Timone, UMR7289, CNRS / Aix-Marseille Université, Marseille, France; Indiana University, UNITED STATES

## Abstract

Due to its inherent neural delays, the visual system has an outdated access to sensory information about the current position of moving objects. In contrast, living organisms are remarkably able to track and intercept moving objects under a large range of challenging environmental conditions. Physiological, behavioral and psychophysical evidences strongly suggest that position coding is extrapolated using an explicit and reliable representation of object’s motion but it is still unclear how these two representations interact. For instance, the so-called flash-lag effect supports the idea of a differential processing of position between moving and static objects. Although elucidating such mechanisms is crucial in our understanding of the dynamics of visual processing, a theory is still missing to explain the different facets of this visual illusion. Here, we reconsider several of the key aspects of the flash-lag effect in order to explore the role of motion upon neural coding of objects’ position. First, we formalize the problem using a Bayesian modeling framework which includes a graded representation of the degree of belief about visual motion. We introduce a motion-based prediction model as a candidate explanation for the perception of coherent motion. By including the knowledge of a fixed delay, we can model the dynamics of sensory information integration by extrapolating the information acquired at previous instants in time. Next, we simulate the optimal estimation of object position with and without delay compensation and compared it with human perception under a broad range of different psychophysical conditions. Our computational study suggests that the explicit, probabilistic representation of velocity information is crucial in explaining position coding, and therefore the flash-lag effect. We discuss these theoretical results in light of the putative corrective mechanisms that can be used to cancel out the detrimental effects of neural delays and illuminate the more general question of the dynamical representation at the present time of spatial information in the visual pathways.

## Author Summary

Visual illusions are powerful tools to explore the limits and constraints of human perception. One of them has received considerable empirical and theoretical interests: the so-called “flash-lag effect”. When a visual stimulus moves along a continuous trajectory, it may be seen *ahead* of its veridical position with respect to an unpredictable event such as a punctuate flash. This illusion tells us something important about the visual system: contrary to classical computers, neural activity travels at a relatively slow speed. It is largely accepted that the resulting delays cause this perceived spatial lag of the flash. Still, after three decades of debates, there is no consensus regarding the underlying mechanisms. Herein, we re-examine the original hypothesis that this effect may be caused by the extrapolation of the stimulus’ motion that is naturally generated in order to compensate for neural delays. Contrary to classical models, we propose a novel theoretical framework, called parodiction, that optimizes this process by explicitly using the precision of both sensory and predicted motion. Using numerical simulations, we show that the parodiction theory subsumes many of the previously proposed models and empirical studies. More generally, the parodiction hypothesis proposes that neural systems implement generic neural computations that can systematically compensate the existing neural delays in order to represent the predicted visual scene at the present time. It calls for new experimental approaches to directly explore the relationships between neural delays and predictive coding.

## Introduction

### Neural delays and motion-induced position shifts

**Fig 1:**
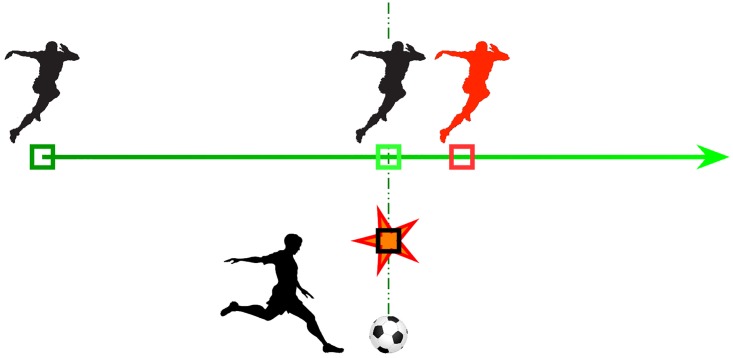
The flash-lag effect (FLE) as a motion-induced predictive shift. To follow the example given by [6], a football (soccer) player that would run along a continuous path (the green path, where the gradient of color denotes the flow of time) is perceived to be ahead (the red position) of its actual position at the unexpected moment a ball is shot (red star) even if these positions are physically aligned. A referee would then signal an “offside” position. Similarly, such a flash-lag effect (FLE) is observed systematically in psychophysical experiments by showing a moving and a flashed stimuli (here, a square). By varying their characteristics (speed, relative position), one can explore the fundamental principles of the FLE.

Though it is barely noticeable in everyday life, visual signals captured on the retina take a significant amount of time before they can elicit even the simplest actions such as eye movements. This neural delay is composed of two terms: a fixed delay caused by the axonal transfer of sensory signals up to motor effectors and a variable delay associated with the neural processing time occurring at each computational step. Moreover, different neural systems can lead to different delays, even for the simplest feed-forward sensorimotor transformations where most of the computational load occurs at sensory level. Just to mention, a delay of 90 ms is observed between the onset of retinal image motion and the first acceleration of tracking eye movements in humans [1–3] while the exact same sensorimotor transformation takes less than 60 ms in monkeys [2]. Furthermore, increasing signal uncertainty would further increase these delays [2] illustrating the fact that neural delays also vary with many environmental or contextual factors. A mere consequence of these unavoidable neural delays should be that we perceive sensory events with a slight, but permanent lag [4, 5]. This is well illustrated in a position estimation task such as the one faced by a soccer referee. If a ball is shot at an unexpected instant by one fixed player, in the direction of another running player, he will generally perceive the moving player “ahead” of its actual position [6] and signal an off-side position despite the fact that the players’ physical positions were strictly aligned to that of the referee (see Fig 1). As a general rule, if no mechanism would intervene to compensate for such neural delays, one would expect severe inefficiencies in sensory computations as well as in goal-directed action control. On the contrary, there are ample evidences that animals can in fact cope with neural delays in order to plan and execute timely goal-directed actions. Thus, it seems evident that throughout natural evolution, some sophisticated compensatory mechanisms based on internal models have been selected [7]. Thus, studying neural delays and how they may be compensated is a critical question that needs to be resolved in order to decipher how basic neural computations such as the dynamical processing of sensory information can be efficiently performed (for a review, see [8]). Solving this enigma would have several theoretical consequences such as, in particular, understanding how neural activity can encode both space and time [9].

Although these neural delays are usually rather short, they can easily be unveiled by psychophysical experiments. This Flash-lag effect (FLE) is a well-studied perceptual illusion which is intimately linked with the existence of neural delays [10]. In a standard empirical variant of the FLE, a first stimulus moves continuously along the central horizontal axis of the screen display. At the time this moving stimulus reaches the center of the screen, a second stimulus is flashed in its near vicinity but in perfect vertical alignment with it. Despite the fact that horizontal positions of the two stimuli are physically identical at the time of the flash, the moving stimulus is most often perceived *ahead* of the flashed one (see the square stimulus in Fig 1). The flash-lag effect falls in the vast category of motion-induced position shifts (e.g. the Fröhlich effect or the representational momentum effect [11–13]), in which the perceived position of an object is biased by its own visual motion or by other motion signals from its visual surrounding. How can we relate the FLE with the existence of the aforementioned neural delays? Several experimental studies have suggested that this visual illusion unveils predictive mechanisms that could compensate for the existing neural delays by extrapolating the object’s motion [4, 14, 15, 13]. Since in natural scenes smooth trajectories are more probable than jittered ones, an internal representation may dynamically integrate information along the trajectory in order to predict the most expected position of the stimulus forward in time, *knowing* an average estimate of the different neural delays. Though computationally simple, this algorithmic solution requires that neural computations can build and use an internal representation of position and velocity over time, that is, that they can manipulate the dynamic representation of a variable.

The aim of our theoretical work is to better understand the interactions between position and motion coding that are based on predictive mechanisms and that could be implemented within the early visual system. To do so, we introduce a generic probabilistic model that was previously shown to efficiently solve other classical problems in sensory processing such as the aperture problem and motion extrapolation [16, 17]. This computational framework allows to quantify the relative efficiency of these different coding mechanisms and to explain the main empirical psychophysical observations. We propose a novel solution for introducing neural delays in the dynamics of probabilistic inference and discuss how this approach is related to previous models of motion diffusion and position coding. Taking the Flash-lag effect as a well-documented illustration of the generic problem of computing with delays, we show that our model can coalesce most of the cardinal perceptual aspects of FLE and thus, unite the previous models described below. More generally, such generic computational principles could be shared by other sensory modalities facing similar delays.

### A brief overview of the Flash-lag effect

The Flash-lag effect was first discovered by Metzger [18] and subsequently investigated by MacKay [10]. After these early studies, the phenomenon did not attract much attention until Nijhawan begun to study a similar question. In his empirical approach, a moving and a static (flashed) stimuli are presented with a perfect spatial and temporal alignment at the time of the flash but most subjects perceive the moving object as leading in space (see Fig 1). Such perceptual effect was reproduced in other species, in particular in monkeys [19]. Motion extrapolation is the correction of the object’s position based on an estimate of its own motion over the time period introduced by neural delays. Nijhawan proposed that such motion extrapolation can explain this perceived spatial offset between the two stimuli. In this theoretical framework, the visual system is predictive and takes advantage of the available information about object’s motion in order to correct for the positional error caused by neural delays.

The seminal work of Nijhawan resurrected the interest for the FLE phenomenon. Since then, the perceptual mechanisms underlying the FLE have been extensively explored by the group of Nijhawan [20–22] and others [23–27] (for a review see [28, 29]). Different variants of the original experiment were designed in order to challenge the different motion extrapolation models. These studies revealed a flaw in Nijhawan’s motion extrapolation theory since it cannot account for the experimental observations made with two specific variants of the FLE, often called half-cycle FLEs [28]. Their common principle is to manipulate the position of the flash relative to the trajectory of the moving object. While in the standard FLE, the flash appears in the middle of the motion path, the flash can now appear either at the beginning or at the end of the motion trajectory, thus defining the flash-initiated and flash-terminated cycle FLEs, respectively. The motion extrapolation hypothesis predicts that, at the beginning of the trajectory, the flashed and moving objects are not likely to be differentiated. However, this prediction was contradicted by the psychophysical results showing a comparable position shift in both the flash-initiated cycle and the standard FLE. Furthermore, extrapolating a trajectory should impose an inertial component even in the presence of sudden changes in the visual motion properties, such as motion termination or reversal. By consequence, the motion extrapolation hypothesis predicts a perceptual overshoot that is similar in both flash-terminated and standard FLE. Again, this prediction was contradicted by psychophysical evidence demonstrating a lack of position shift in the flash-terminated cycle FLE [27]. Lastly, several studies suggested that the motion extrapolation hypothesis needs to be supplemented with complementary mechanisms such as the a posteriori correction of the predicted position, in order to account for the perceived position after an abrupt change in the motion trajectory [30–32].

These new empirical evidences called for alternative hypotheses able to unify all of these different aspects of FLE. A first set of studies proposed that moving and static objects are processed with different latencies in the early visual system. Hence, the perceived lag in FLE could be explained by the faster processing of moving objects, as compared to flashed inputs [23, 33, 24, 15]. There may exist multiple origins at retinal and cortical levels for a more rapid processing of moving objects. Some authors reasoned that, since both flashed and moving stimuli are processed and transmitted within a single (magno-cellular) retino-thalamo-cortical pathway, any difference would be explained by intra-cortical mechanisms that would process differently predictable and unpredictable events [15]. However, there is still a lack of solid neurophysiological empirical evidences in support of this differential latency hypothesis. A second hypothesis suggested that the FLE may be explained by the position persistence for the flashed visual input [34, 35]. The central idea is that motion information is averaged within a 500 ms window. By consequence, the perceived position of the flash would persist, while the averaged position for the moving object is perceived ahead of its actual position, along its motion path. The main flaw of this hypothesis is that the supposed time constant (500 ms) is unrealistically long with respect to the known dynamics of motion integration.

More recently, Wojtach et al. [36] proposed that the FLE may be seen as a mere consequence of the distribution of speeds that are empirically observed during our visual experience. Using the perspective transform from the three-dimensional physical space to the two-dimensional plane of the retinotopic space, they assigned empirical probabilities of the observed retinal speeds from the mapping of objects’ velocities in the physical world. By doing so, they defined an *a priori* probability distribution of speeds which can be combined with sensory evidence. This solution proposes a probabilistic framework inferring an optical estimate of motion speed. Such estimate is then used in a motion extrapolation model compensating for the neural delay. The authors estimated the amplitude of the lag in respect to an extended speed range of object motion. Their model depicts a nonlinear relationship between motion speed and the perceptual lag, similar to the one observed with the standard flash-lag experiment. Thus, the model from Wojtach et al. [36] provides an ingenious extension of the motion extrapolation model using inferred speed. However, this model was not tested against the aforementioned complete, and challenging set of empirical studies probing the FLE at different epochs of the motion trajectory.

One last approach is the *postdiction* hypothesis [27] postulating that visual awareness attributes the position of a moving stimuli at the instant of the flash appearance according to the information collected within an ≈80ms time window following the flash. In particular, the flash is considered as a reset for motion integration and, as such, this would be sufficient in explaining why the FLE is not perceived in the flash-terminated cycle. The postdiction hypothesis relies on two main assumptions. First, both moving and flashed inputs have the same neural delays. Second, the flash acts as a resetting mechanism. By consequence, the model predicts that observers shall perceive both a spatial lag of the flash and a change in the speed of the moving object. However, such a speed increment has never been reported in the context of FLE [28]. The postdiction model is thus an elegant hypothesis that allows us to understand a wide range of variants of the FLE but fails to explain this later aspect of FLE. In summary, the half-cycles variants of the FLE introduced by Eagleman and Sejnowski [27] remain challenging for all current theoretical approaches of the FLE, despite the fact that they might reveal how the visual system processes motion onset and offset and their impact on position coding.

### The parodiction hypothesis

Overall, previous theoretical studies can be grouped according to two main hypotheses. On one hand, models based on latency difference or motion extrapolation rely on how the neural representation of position information is encoded. On the other hand, the postdiction hypothesis is based on how visual awareness decodes objects’ positions from neural activity in a short temporal window. In the present theoretical study, we will propose a new hypothesis which subsumes both of these aspects. Our theoretical approach is based upon two major constraints faced by any neural system, in comparison to a conventional computer, when estimating the position of an object: First, there is no access to a central clock, that is, the present, exteroceptive, physical timing is hidden (or latent in machine learning terms) to the nervous system. Second, the internal representation encoded in the neural activity is distributed and dynamical. In particular, the system is confronted to non-stationary sources of noises and has to provide for an optimal estimate at any time for upstream neural networks.

Driven by these constraints, a biologically-realistic hypothesis is that a perceived position corresponds to the most likely position at the present time [37]. According to the probabilistic brain hypothesis (see [8] for a generic application to eye movements), an optimal solution is that the internal representation encodes beliefs in the form of probability distribution functions (pdf) and that the optimal estimate is computed knowing both the instantaneous sensory data and the internal representation. When the represented variable, such as the position, is predictable, this process involves that the internal representation uses a generative model of its dynamics to progressively refine the estimation. As a result, using a probabilistic formulation of predictive coding, it is possible to explicitly represent the instantaneous information about object’s motion and its precision, coherently with the role played by perceptual precision in the FLE [38]. Consequently, we propose that a generic goal of these neural computations is to optimally align the position represented in the neural activity with that at the veridical, but hidden, physical time. We will call this approach the *parodiction* hypothesis, from the ancient Greek *παρóν*, the present time.

Herein, we will show that probabilistic motion extrapolation can efficiently compensate for the neural delays and explain the shift in perceived positions in the different variants of the FLE. The paper is organized as follows. First, we will define the probabilistic motion extrapolation model and we will describe how delays can be taken into account. This model extends a simple motion-based predictive model based on the temporal coherency of visual trajectories that we proposed earlier [16] and is also a novel formulation of the original motion extrapolation model proposed by Nijhawan [4]. Second, we present the results of this model with the standard FLE and in particular we titrate the role of the velocity of the moving object. Then, we will show that the model can account for both standard and half-cycle FLEs. In particular, we will show that within this optimal integration scheme, the relative precision of sensory and internal information may modulate the gain of their interaction. This is first illustrated by challenging the model with a motion reversal experiment. To further investigate this behavior, we manipulated the contrast of the stimuli. This allowed us to dynamically switch the system from a purely feed-forward model, exhibiting differential latencies, to a model showing a pure motion extrapolation behavior. We will finally discuss the advantages and limitations of our *parodiction* hypothesis, in comparison with the previously proposed models.

## Methods

### Motion-based prediction and the diagonal models

This computational study explores the potential role of predictive coding in explaining the dynamics of position coding. Similar to most predictive models, a natural choice for the representation of information is to use probabilities. Thus, the motion of an object is best described by the probability distribution function (pdf) of its instantaneous position (*x*, *y*) and velocity (*u*, *v*) [16]. Note that these coordinates are defined in the planar visual space, under the assumption that we model small displacements in the vicinity of the visual axis. The pdf *p*(*x*, *y*, *u*, *v*) represents at a given time the degree of belief among a set of possible positions and velocities. In this framework, a Bayesian predictive model will optimally integrate the sensory information available from the sensory inputs (likelihood) with an internal model (i. e. an *a priori* distribution) of state transition in order to compute an *a posteriori* pdf of motion. Typically, the likelihood is computed using a model of sensory noise, an approach that fits well to the motion energy model of the direction-selective cells in the cortex [39–41]. By sequentially combining at any given time *t* the *a posteriori* estimate with the likelihood using the prior on state transition, we implement a Markov chain forming a dynamical predictive system.

One novelty of motion-based prediction is to encapsulate the coherency of motion trajectory in the internal model, that is, in the prior of state transition. In particular, this prior knowledge instantiates a preference for smooth transitions of successive motion states, as expected from the statistics of natural visual motion trajectories [40]. Such an internal model was first proposed in a neural network implementing the detection of a single moving dot embedded in very high level of noise [42]. More recently, we have shown that this motion-based prediction model can explain the dynamics of the neural solution to both the aperture problem [16] and motion extrapolation [17]. In the present study, we will show that it can also be used to compensate for known neural delays [8]. It is important to recall that our model is reminiscent of the diagonal model originally proposed by [21] (called thereafter Nijhawan’s diagonal model), but with one important distinction: motion information is now represented by probabilities.

### A probabilistic implementation of the Nijhawan’s diagonal model

In order to define a predictive system, one can use a classical Markov chain formed by sequentially combining, at any given time, the likelihood with a prior on state transition. When describing visual motion (i.e., position and velocity) at time *t* by the instantaneous state vector *z*_*t*_ = (*x*_*t*_, *y*_*t*_, *u*_*t*_, *v*_*t*_), the master equations of this Markov chain become:

$$\text{estimation}:\;p\left(z_{t}|I_{0:t}\right)\propto p\left(I_{t-\delta t:t}|z_{t}\right)\cdot p\left(z_{t}|I_{0:t-\delta t}\right)$$ (1)

$$\text{prediction}:\;p\left(z_{t}|I_{0:t-\delta t}\right)=\int dz_{t-\delta t}\cdot p\left(z_{t}|z_{t-\delta t}\right)\cdot p\left(z_{t-\delta t}|I_{0:t-\delta t}\right)$$ (2)

where *p*(*I*_*t* − *δt*:*t*_|*z*_*t*_) is the likelihood computed over the infinitesimally small temporal window [*t* − *δt*, *t*), that is, the time window of width *δt* before present time *t*. By definition, the pdf *p*(*z*_*t*_|*I*_0:*t*_) corresponds to the belief in the motion *z*_*t*_ at time *t*, knowing the sensory information *I* being integrated over the temporal window starting at the beginning of the observations (*t* = 0) and extending to the current time *t*. Notice that the probabilistic notations will allow us to conveniently describe the current belief on the state vector before observing a new sensory information as the pdf *p*(*z*_*t*_|*I*_0:*t*−*δt*_). Importantly, this dynamical system is biologically realistic as it describes the belief in a macroscopic time window [0, *t*), based only on the integration of the information available at the present time [*t* − *δt*, *t*).

Intuitively, this model combines the two basic operations of probability theory. First, in the estimation stage, the multiplication corresponds to the combination of two independent pieces of information: one element is derived from the measurements (i.e. the likelihood *p*(*I*_*t* − *δt*:*t*_|*z*_*t*_)) and the other is the current knowledge about the state before the measurements. Information is assumed to be conditionally independent because the source of noise in the likelihood (measurement noise) is assumed to be independently generated from the internally generated state estimation noise. This first stage corresponds to a AND operator in boolean logic. Second, in the prediction state, the possible state *p*(*z*_*t*_|*I*_0:*t*−*δt*_) is inferred by an addition over all possible previous states, given by the integral sign. The integrals sum over the whole distribution of estimated positions at *t* − *δt* the possible state transitions that would yield *z*_*t*_. By consequence, very unlikely states (at time *t* − *δt*) and state transitions (for instance, incoherent non-smooth motions) will have little weight in this summation. This computational step corresponds to a OR operator in boolean logic. These two steps implement the classical “predict-update cycle” of the Markov model and are sufficient to define our dynamical predictive coding model.

**Fig 2:**
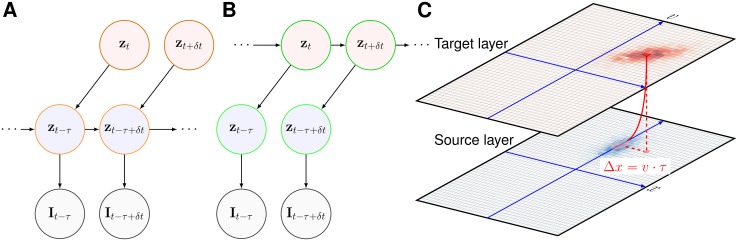
Diagonal Markov chain. In the current study, the estimated state vector *z* = {*x*, *y*, *u*, *v*} is composed of the 2D position (*x* and *y*) and velocity (*u* and *v*) of a (moving) stimulus. (A) First, we extend a classical Markov chain using Nijhawan’s diagonal model in order to take into account the known neural delay *τ*: At time *t*, information is integrated until time *t* − *τ*, using a Markov chain and a model of state transitions *p*(*z*_*t*_|*z*_*t*−*δt*_) such that one can infer the state until the last accessible information *p*(*z*_*t*−*τ*_|*I*_0:*t*−*τ*_). This information can then be “pushed” forward in time by predicting its trajectory from *t* − *τ* to *t*. In particular *p*(*z*_*t*_|*I*_0:*t*−*τ*_) can be predicted by the same internal model by using the state transition at the time scale of the delay, that is, *p*(*z*_*t*_|*z*_*t*−*τ*_). This is virtually equivalent to a motion extrapolation model but without sensory measurements during the time window between *t* − *τ* and *t*. Note that both predictions in this model are based on the same model of state transitions. (B) One can write a second, equivalent “pull” mode for the diagonal model. Now, the current state is directly estimated based on a Markov chain on the sequence of delayed estimations. While being equivalent to the push-mode described above, such a direct computation allows to more easily combine information from areas with different delays. Such a model implements Nijhawan’s “diagonal model”, but now motion information is probabilistic and therefore, inferred motion may be modulated by the respective precisions of the sensory and internal representations. (C) Such a diagonal delay compensation can be demonstrated in a two-layered neural network including a source (input) and a target (predictive) layer [44]. The source layer receives the delayed sensory information and encodes both position and velocity topographically within the different retinotopic maps of each layer. For the sake of simplicity, we illustrate only one 2D map of the motions (*x*, *v*). The integration of coherent information can either be done in the source layer (push mode) or in the target layer (pull mode). Crucially, to implement a delay compensation in this motion-based prediction model, one may simply connect each source neuron to a predictive neuron corresponding to the corrected position of stimulus (*x* + *v* ⋅ *τ*, *v*) in the target layer. The precision of this anisotropic connectivity map can be tuned by the width of convergence from the source to the target populations. Using such a simple mapping, we have previously shown that the neuronal population activity can infer the current position along the trajectory despite the existence of neural delays [44].

However, in this mathematical framework, one needs to gain an immediate access to sensory information, that is, to know the image *I* at time *t*, in order to compute *p*(*I*_*t*−*δt*: *t*_|*z*_*t*_). This is impossible in the presence of neural delays. Considering a known (fixed) neural delay *τ*, at time *t* the system only had access to *I*_0:*t*−*τ*_ and thus one needs to estimate *p*(*z*_*t*_|*I*_0: *t*−*τ*_). An essential property of motion-based predictive coding is the ability to extrapolate motion when sensory information is transiently absent [17]. As illustrated in Fig 2-A, the probability distribution function *p*(*z*_*t*_|*I*_0:*t*−*τ*_) may be predicted by “pushing forward” the information *p*(*z*_*t*−*τ*_|*I*_0:*t*−*τ*_) such as to compensate for the delay, while being still recursively computed in a way similar to a classical Markov chain model (see the bottom part of Fig 2-A). Thus, a classical Markov chain in the presence of a known delay can be redrawn in a “diagonal” mode. It is similar to the original suggestion made by Nijhawan and Wu [21] in order to explain the detailed mechanism of motion extrapolation in retinal ganglion cells. Here, we generalize this diagonal mode as a probabilistic model of predictive motion estimation.

As a consequence, the master equations of this diagonal model can be written as:

$$\text{estimation}:\;p\left(z_{t-\tau }|I_{0:t-\tau }\right)\propto p\left(I_{t-\tau -\delta t:t-\tau }|z_{t-\tau }\right)\cdot p\left(z_{t-\tau }|I_{0:t-\tau -\delta t}\right)$$ (3)

$$\text{prediction}:\;p\left(z_{t-\tau }|I_{0:t-\tau -\delta t}\right)=\int dz_{t-\tau -\delta t}\cdot p\left(z_{t-\tau }|z_{t-\tau -\delta t}\right)\cdot p\left(z_{t-\tau -\delta t}|I_{0:t-\tau -\delta t}\right)$$ (4)

$$\text{extrapolation}:\;p\left(z_{t}|I_{0:t-\tau }\right)=\int dz_{t-\tau }\cdot p\left(z_{t}|z_{t-\tau }\right)\cdot p\left(z_{t-\tau }|I_{0:t-\tau }\right)$$ (5)

As evidenced by these equations, Eqs 3 and 4 are similar to Eqs 1 and 2, except that these are now delayed by *τ*, the known sensory delay. This information *p*(*z*_*t*−*τ*_|*I*_0:*t*−*τ*_) is then “pushed” forward in time using the extrapolation step (see Eq 5), in a similar fashion to the predictive step on the infinitesimal period (Eqs 2 and 4) but now on the possibly longer period of the sensory delay (in general *τ* ≫ *δt*). As a result, we obtain the estimate of motion at the current time, knowing the information acquired until *t* − *τ*, that is, *p*(*z*_*t*_|*I*_0:*t*−*τ*_). Finally, the next states correspond to the integration of the estimations at the actual current stimulus position and motion, overcoming the restrictive effect of delay [8]. Note that the earliest part of the trajectory is necessarily missed since motion estimation begins integrating sensory information only after the delay *τ*, as there is no sensory input before. Decisively, this model is now compatible with our initial hypothesis that sensory information is only available after a delay.

Although this first model (i.e. the “pushing” mode) is the easiest to understand with respect to a Markov chain, it is less practical to consider within a biological setting since it defines that, at time *t*, the state is inferred from a representation of a past state *p*(*z*_*t*−*τ*_|*I*_0:*t*−*τ*_). For neural networks implementations, the internal representation (as encoded by the neural activity) is only accessible at the present time. As a consequence, it may be more convenient to derive a set of predictive steps that would directly act on the estimation of the state at the current time *p*(*z*_*t*_|*I*_0: *t*−*τ*_). This question is particularly acute for complex architectures mimicking the deep hierarchies of low-level visual cortical areas where information should not be conditioned by the delays arising at each processing layer but rather be based on a common temporal reference such as the current time *t*. In that objective, one notes that, by merging the estimation and prediction steps in the master equation, we obtain:

$$\begin{array}{cc} p(z_{t}|I_{0:t-\tau })= & \int dz_{t-\tau }\cdot p\left(z_{t}|z_{t-\tau }\right)\cdot p\left(z_{t-\tau }|I_{0:t-\tau }\right)\\ \propto & \int dz_{t-\tau }\cdot p\left(z_{t}|z_{t-\tau }\right)\cdot \left[p\left(I_{t-\tau -\delta t:t-\tau }|z_{t-\tau }\right)\cdot p\left(z_{t-\tau }|I_{0:t-\tau -\delta t}\right)\right]\\ \propto & \left[\int dz_{t-\tau }\cdot p\left(z_{t}|z_{t-\tau }\right)\cdot p\left(I_{t-\tau -\delta t:t-\tau }|z_{t-\tau }\right)\right]\cdot \int dz_{t-\tau -\delta t}\cdot p\left(z_{t-\tau }|z_{t-\tau -\delta t}\right)\cdot \end{array}$$ (6)

$$p(z_{t-\tau -\delta t}|I_{0:t-\tau -\delta t})$$ (7)

Regrouping terms, it becomes:

$$p\left(z_{t}|I_{0:t-\tau }\right)\propto \int dz_{t-\tau }\cdot p\left(z_{t}|z_{t-\tau }\right)\cdot \left[\int dz_{t-\tau -\delta t}\cdot p\left(z_{t-\tau }|z_{t-\tau -\delta t}\right)\cdot p\left(I_{t-\tau -\delta t:t-\tau }|z_{t-\tau }\right)\cdot p\left(z_{t-\tau -\delta t}|I_{0:t-\tau -\delta t}\right)\right]$$ (8)

The term within brackets can be written as an argument of an extrapolation from *t* − *τ* to *t*, yielding to:

$$p\left(z_{t}|I_{0:t-\tau }\right)\propto \int dz_{t-\delta t}\cdot p\left(z_{t}|z_{t-\delta t}\right)\cdot p\left(I_{t-\tau -\delta t:t-\tau }|z_{t}\right)\cdot p\left(z_{t-\delta t}|I_{0:t-\tau -\delta t}\right)$$ (9)

As frequently assumed, the transition matrix is stationary: our prior assumption on the internal model (here, the parameters with which we model the coherence of trajectories) do not change over time. Finally, regrouping terms, we obtain:

$$p\left(z_{t}|I_{0:t-\tau }\right)\propto p\left(I_{t-\tau -\delta t:t-\tau }|z_{t}\right)\cdot \left[\int dz_{t-\delta t}\cdot p\left(z_{t}|z_{t-\delta t}\right)\cdot p\left(z_{t-\delta t}|I_{0:t-\tau -\delta t}\right)\right]$$ (10)

Therefore, the master equation to the “push” mode are equivalent to:

$$\text{estimation}:\;p\left(z_{t}|I_{0:t-\tau }\right)\propto p\left(I_{t-\tau -\delta t:t-\tau }|z_{t}\right)\cdot p\left(z_{t}|I_{0:t-\tau -\delta t}\right)$$ (11)

$$\text{prediction}:\;p\left(z_{t}|I_{0:t-\tau -\delta t}\right)=\int dz_{t-\delta t}\cdot p\left(z_{t}|z_{t-\delta t}\right)\cdot p\left(z_{t-\delta t}|I_{0:t-\tau -\delta t}\right)$$ (12)

$$\text{extrapolation}:\;p\left(I_{t-\tau -\delta t:t-\tau }|z_{t}\right)=\int dz_{t-\tau }\cdot p\left(z_{t}|z_{t-\tau }\right)\cdot p\left(I_{t-\tau -\delta t:t-\tau }|z_{t-\tau }\right)$$ (13)

We will call this second mode the “pulling” mode and is illustrated in Fig 2-B.

The two modes that have been presented above share the same processing logic but have different implications about the manner with which both the internal model and the likelihood function might be implemented. In the pushing mode, the motion state *z*_*t*−*τ*_ is estimated from both a delayed sensory input *I*_*t*−*τ*−*δt*:*t*−*τ*_ and the motion coherency. Eq 3 calculates the probability of a desired motion state, using the likelihood of that state (measured from the sensory information with a delay *τ*) and the predicted belief given by Eq 4. At the next step, the estimated motion is extrapolated for a period of duration *τ*, similar to a “virtual blank” during which there is no sensory measurements [17]. Thus, the extrapolation step shown by Eq 5 is purely predictive, under the constraint of motion coherency (see Eq 12) and with the available information about the delay *τ*. In the pulling mode, the probabilistic representation is different as the current state is directly estimated from the delayed measurements and the extrapolative step is “hidden” in the probability *p*(*z*_*t*_|*I*_0:*t*−*τ*−*δt*_). Under the stationarity assumption, both modes are mathematically equivalent and produce the same probabilistic representation of instantaneous states based on delayed measurements.

In summary, the information about the motion estimates (position, velocity) at time *t* knowing the sensory information observed between 0 and *t* − *τ* is contained in the pdf *p*(*z*_*t*_|*I*_0:*t*−*τ*_). As we have seen above, it can be computed using the diagonal model in push mode and summarized in the following master equations:

$$p\left(z_{t}|I_{0:t-\tau }\right)\propto \int dz_{t-\delta t}\cdot p\left(z_{t}|z_{t-\delta t}\right)\cdot p\left(I_{t-\tau -\delta t:t-\tau }|z_{t}\right)\cdot p\left(z_{t-\delta t}|I_{0:t-\tau -\delta t}\right)$$ (14)

$$p\left(I_{t-\tau -\delta t:t-\tau }|z_{t}\right)=\int dz_{t-\tau }\cdot p\left(z_{t}|z_{t-\tau }\right)\cdot p\left(I_{t-\tau -\delta t:t-\tau }|z_{t-\tau }\right)$$ (15)

Eqs 14 and 15 are the master equations of Nijhawan’s diagonal model when framing it in a probabilistic setting. Importantly, the inferred motion may be modulated by the respective precisions of the sensory (*p*(*I*_*t*−*τ*−*δt*:*t*−*τ*_|*z*_*t*_)) and internal (*p*(*z*_*t*−*δt*_|*I*_0: *t*−*τ*−*δt*_)) representations. The model gives a probabilistic distribution of the estimated motion state *z*_*t*_, based on delayed motion measurements *I*_*t*−*τ*_. In the next section, we will describe how the transition probability distribution functions *p*(*z*_*t*_|*z*_*t*−*τ*_) and *p*(*z*_*t*_|*z*_*t*−*δt*_) are computed.

### Diagonal motion-based prediction (dMBP)

We have seen above that one needs to characterize the temporal coherency of motion for different temporal steps, as represented by *p*(*z*_*t*_|*z*_*t*−Δ*t*_) with Δ*t* = *δt* or Δ*t* = *τ*. Assuming that motion is *transported* in time during this time period of Δ*t* (with a drift similar to a Brownian motion and characterized by some given diffusion parameters), we obtain [16]:

$$\left\{\begin{array}{cc} \;x_{t} & =x_{t-\Delta t}+u_{t-\Delta t}\cdot \Delta t+\nu_{x}\\ \;y_{t} & =y_{t-\Delta t}+v_{t-\Delta t}\cdot \Delta t+\nu_{y} \end{array}\right.$$ (16)

$$\left\{\begin{array}{cc} \;u_{t} & =\gamma \cdot u_{t-\Delta t}+\nu_{u}\\ \;v_{t} & =\gamma \cdot v_{t-\Delta t}+\nu_{v} \end{array}\right.$$ (17)

Here, $\gamma ={\left(1+\frac{D_{V}}{\sigma_{p}^{2}}\right)}^{-1}$ is the damping factor introduced by the prior on slowness of motion [17]. As defined by Weiss and Fleet [40], this prior information about slowness and smoothness of visual motion can be parameterized by its variance $\sigma_{p}^{2}$ and *γ* ≈ 1 for a high value of *σ*_*p*_.

The diffusion parameters characterize the precision of the temporal motion coherency and are parameterized by the variance of the Gaussian distributions that define the additive noise *ν*_*x*_, *ν*_*y*_ in the transport equations. First, the variance *D*_*X*_ ⋅ |Δ*t*| setting the blur in position define the noise distribution as:

$$\nu_{x},\nu_{y}\propto N(\left(x,y\right);\left(0,0\right),D_{X}\cdot \left|\Delta t|\right)$$ (18)

where $N(\left(x,y\right);\mu ,\sigma^{2})$ is the two dimensional normal distribution on real numbers *x* and *y* of mean $\mu \in R^{2}$ and variance *σ*^2^ (assuming isotropy of the noise, the covariance matrix is unitary). Concerning velocity, this models assumes similarly that *ν*_*u*_ and *ν*_*v*_ are modeled by Gaussian distributions:

$$\nu_{u},\nu_{v}\propto N(\left(u,v\right);\left(0,0\right),{\left(\sigma_{p}^{-2}+D_{V}^{-1}\right)}^{-1}\cdot \left|\Delta t|\right)$$ (19)

where the diffusion parameter *D*_*V*_ parameterizes the dynamics of the motion vector. Finally, the variance equals to ${\left(\sigma_{p}^{-2}+D_{V}^{-1}\right)}^{-1}\cdot \left|\Delta t\right|$. Note that for a very weak prior for slow speeds, $\sigma_{p}^{-2}\approx 0$ and ${\left(\sigma_{p}^{-2}+D_{V}^{-1}\right)}^{-1}\approx D_{V}$ such that it is then similar to the Brownian diffusion equation on position. This updating rule (see [41] for a derivation) assumes independence of the prior on slow speeds with respect to predictive prior on smooth trajectories (see [17] for more details).

From these generative equations, one may then compute both *p*(*z*_*t*−*τ*_|*z*_*t*−*τ*−*δt*_) and *p*(*z*_*t*_|*z*_*t*−*τ*_) using Eqs 16 and 17 and the assumption in Eqs 18 and 19. Note also that in all generality, we have used a formulation where extrapolation can be performed forwards in time (Δ*t* > 0), but also backwards (Δ*t* < 0), as it may be useful in some cases to guess a position in a past state, when only knowing the state at the present time. By including the compensation for the neural delay in the motion-based prediction (MBP) model, this defines a novel, more general diagonal model that we call diagonal motion-based prediction (dMBP).

### A neural interpretation of the dMBP model

From a biological perspective, it seems very unlikely that sensory neurons can store complex time series about past variables. A strong constraint in understanding the biologically-plausible mechanisms for delay compensation is to build models which would only use the neural activity available at the present time. As such, one solution may arise from generalized representation of variables where a given variable (e.g. position) is represented at any given time *t* by its value and its instantaneous time derivatives (e.g. velocity, acceleration and so on). In a previous computational study on the compensation of delays in neural networks [8], we introduced the idea that sensory delays can be (internally) simulated and corrected by applying delays to sensory inputs producing sensory prediction errors. In a biologically-realistic network, the application of delay operators can be implemented by changing synaptic connection strengths in order to capture different mixtures of generalized sensations and their prediction errors. The precision of this compensation (and therefore on the range of delays it can compensate) is highly dependent upon the number of orders in the generalized representation and on their respective precision, as measured by the inverse of their variance (for a detailed mathematical account, see [43]). In other words, in a neural network encoding both position and velocity, a compensation for delays in the sort we described above may be easily achieved solely by appropriately setting the matrix of connectivity weights.

The present motion-based predictive coding can be implemented in a simple two-layered neural network as illustrated in Fig 2-C. The source layer implements a neural representation of sensory inputs and activates specific populations of the target layer. More specifically, the neural activity within the input layer represents the likelihood of the sensory input at time *t* knowing the delay *τ*, that is, *p*(*I*_*t*−*τ*−*δt*:*t*−*τ*_|*z*_*t*_). In particular, the mapping between the source and target layers is fixed but anisotropic (as it is implementing *p*(*z*_*t*_|*z*_*t*−*τ*_)), the bias depending upon the estimated velocity and neural delay *τ*. For instance, in the case of the example neuron displayed in Fig 2-C, its efferents in the target layer may be interpreted as a neural population which is stimulated by the sensory information received by some “rightward” neurons centered on its left. Finally, a third stream of information allows to update the dynamics of the internal model (that is, of *p*(*z*_*t*_|*I*_0: *t*−*τ*_)) using lateral connectivity. From the mathematical equivalence between the push mode and the pull mode that was presented above, this could be implemented indifferently in the source layer by implementing *p*(*z*_*t*−*τ*_|*z*_*t*−*τ*−*δt*_) or in the target layer by implementing *p*(*z*_*t*_|*z*_*t*−*δt*_). It shall be noticed that the architecture of this model is not fundamentally different from our previous model of motion extrapolation during a transient blanking of the sensory flow [17]. Now the sensory delay consists in a “virtual blank” during which sensory information is not sensed. Since the architecture of our previous model has been already implemented in a spiking neural network (SNN) using anisotropic connectivity patterns [44], one could easily provide a similar neural network implementation of this dMBP model.

Once the dMBP model is defined, it is important to briefly highlight its analogies with other implementations. For instance, instead of considering a parametric model for the prior distributions, we may use an empirical prior, such as the one defined for speed by Wojtach et al. [36]. It is however important to note that a departure of our model with that of Wojtach et al. [36] is the fact that, instead of using the inferred speed from the likelihood and the speed prior, we use the probability distribution function for the representation of motion. In particular, the precision of the information in the source and target layers will be essential to weight the dynamical integration of information during the flow of sensory information. This proved to be essential in a dynamical display such as the FLE. More generally speaking, when the sensory input is best described by a Gaussian distribution, our model is equivalent to a Kalman filter, in the form of an optimal smoother, as previously introduced by Rao et al. [45] (see also [8] for a more rigorous and extended mathematical formulation). However, this latter model used a sequential representation of the activity both in external (physical) and internal temporal spaces. There is no neurophysiological evidence that such a representation could be implemented. Rather, we reasoned that all information should be available at the present time.

## Results

We tested our model with the different instances of the FLE conditions and manipulated the parameters of the static (flashed) and moving stimuli in order to explore the advantages of motion-based position coding with respect to previous models. The dMBP model was implemented with a particle filter method which has been previously detailed in [16]. An extensive parameter scan for simulations was performed on a cluster of Linux nodes (Debian Wheezy) using python (version 3.5.0) and numpy (version 1.10.1). The code written to produce all figures and supplementary materials is available on the corresponding author’s website at http://invibe.net/LaurentPerrinet/Publications/KhoeiMassonPerrinet17. On a standard laptop computer (early 2015 MacBook pro Retina with 3,1 GHz Intel Core i7 and 16 GB DDR3 memory), a video of resolution 256 × 256 at 100 frames per second is approximately processed at half real time such that reproducing all the figures presented herein takes approximately one hour of processing.

The model and its simulations are controlled by a limited set of parameters. The MBP model originally described in [16] was controlled only by the 2 diffusion parameters (*D*_*X*_ and *D*_*V*_), the width of the slow speed prior and, lastly the parameters of the motion energy model used to estimate the likelihoods (that is, the estimate of background noise’s variance and a gain element). In particular, the likelihood is computed using a motion energy based on a generative model of the motion of objects in natural scenes [46]. We used the same values as in [16], which have been shown as yielding to the emergence of a stable tracking behavior when presented with a smooth rectilinear trajectory (see Table 1). The present extension of this MBP model to the current dMBP model adds a single new parameter, the sensory latency *τ*.

**Table 1:** Model’s parameters. The model is implemented using the same paradigm as detailed in [16] while the extrapolation uses the same formalism as in [17]. We used a similar set of parameters and controlled by a set of iPython notebooks that are available on the corresponding author’s website. Note that the speed and diffusion parameters are given relative to one spatio-temporal period.

Name	Value	Role	Range
*D*_*X*_	1	diffusion parameter in position	$R^{+}$
*D*_*V*_	1	diffusion parameter in speed	$R^{+}$
*σ*_*p*_	3	characteristic value for the speed prior	$R^{+}$
*N*_*T*_, *δ*_*t*_	100, *T*/*N*_*T*_ = .01	number of frames, time step (in seconds)	$Z^{+}$
*τ*	10/*N*_*T*_ = .1	fixed delay (latency, in seconds)	$Z^{+}$
*N*_*X*_, *N*_*Y*_	256, 256	number of pixels	$Z^{+}$
*σ*_*I*_	.25	standard deviation in the motion energy model	$R^{+}$
*σ*_*noise*_	0.05	standard deviation of the assumed noise	$R^{+}$

Finally, the parameters of the visual stimulation were the speed and length of the moving dot trajectory, as well as the position and duration of the flash (see Table 2). To efficiently titrate the role of these parameters, we built a computational framework to test different parameters ranges. This code is available as a collection of iPython notebooks which allow to reproduce each figure of this paper but also to track the role of each respective parameter. In particular, results with different FLE conditions (i.e. short flashes, dot visibility after motion stop…) were qualitatively similar. The respective contributions of both model and visual stimuli parameters will be described below.

**Table 2:** Stimuli’s parameters. Stimuli are generated on a space defined in absolute values (ranging arbitrarily from −1 to 1) and time defined from *t* = 0 to *t* = *T* (in seconds). As such, stimulus parameters are defined in these units. To avoid border effects, the spatio-temporal domain is defined as a 3-dimensional torus (that is the cartesian product of the periodic real spaces R/2Z⊗R/2Z⊗R/TZ). By convention, a speed of 1 is defined as a motion of one spatial period in one temporal period.

Name	Value	Role	Range
*T*	1	duration (in seconds) of the stimuli)	$R^{+}$
*dot*_*size*_	0.05	size of the dot	$R^{+}$
*V*	1	speed of the dot	R
*dot*_*start*_	.2	start of trajectory (in seconds)	[0, *T*]
*dot*_*stop*_	.8	end of trajectory (in seconds)	[*dot*_*start*_, *T*]
*I*_*noise*_	.05	std of noise in images	$R^{+}$
*T*_*f*_	0.05	flash duration (in seconds)	$R^{+}$

### Diagonal motion-based prediction (dMBP) and the flash-lag effect (FLE)

The standard FLE experiment is composed of a simple moving stimulus (a dot) and a static flash that appears in perfect alignment as the stimulus crosses the middle of its trajectory (Fig 1). We reproduced these exact conditions to test our model. Defining the spatial coordinates between −1 and 1, the dot started at *t* = 0.2 s for rightward motion from *x* = −0.6, moved toward *x* = 0.6 and then disappeared at this position. Thanks to the symmetry between left- and rightward motions, we will only show the results for the pure horizontal rightward motion of a small dot. We defined an absolute time in arbitrary units for which motion begun at *t* = 200 ms and ended at *t* = 800 ms. In the simulations, this period of time was subdivided into 100 frames such that every frame of stimulus was arbitrarily set to 10 ms of biological time and one temporal period lasted 1 second. In all experiments, the flash persisted for 5% of display duration (5 frames out of 100, that is, frames #48 to #52). Notice that such flash duration is much longer than the microsecond duration used in psychophysical experiments [16]. Such ultrashort duration was set to avoid retinal persistence and other perceptual effects that could interact with the perceived timing but was irrelevant for the current modeling study. Still, we run the model with a range of these timing values and checked that the results remain qualitatively similar. Lastly, all results were computed over 20 independent repetitions by changing the seed of the number generator that governs the generation of sensory and internal noise.

**Fig 3:**
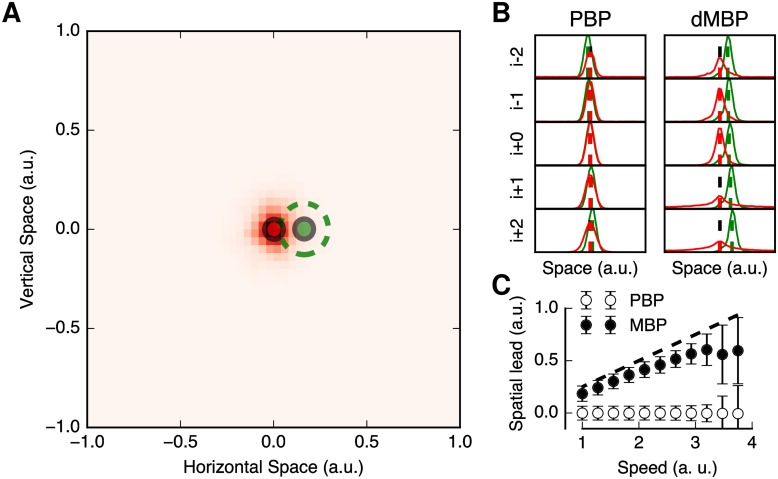
The diagonal motion-based prediction (dMBP) model accounts for the Flash-lag effect. (A) We plot the histogram of estimated positions from the dMBP model with a neural delay *τ* = 100 ms for the moving and the flashed stimuli. These estimated positions are averaged across the five frames centered around the time at which the response to the flash reaches its maximal precision and across 20 trials. Comparing the distribution of estimated positions for the moving (green) and flashed (in red) stimuli shows that, at this particular instant, the (left) moving dot is perceived ahead of the estimated position of the flash. (B) We quantified this spatial lead by plotting the histograms of the inferred horizontal positions during these frames, both for the position-based predictive (PBP) and dMBP models. The red and green dashed vertical lines represent the average positions of the flashed and moving stimuli, respectively. One can observe a significant spatial lead in the dMBP model, but not in the PBP model. The motion component of the dMBP model is thus essential to explain the flash-lag effect. (C) We varied the speed of the dot motion to titrate its role in the amplitude of the spatial lead. The black dashed line illustrates the predicted linear relationship from an extrapolation model with a perfect knowledge about target speed (slope one). One can observe a nearly linear relationship at slow speeds, followed by a saturation for higher speeds. At the fastest extrema of the speed range, ones observes a decrease in the spatial lead of the moving spot, together with an higher variability across trials (error bars: ±1 SD), consistent with the experimental data from [36]. The nonlinear relationship in our model emerges from the decrease of precision in the representation of motion at higher speeds. It highlights the putative role of the dynamic, explicit representation of precision in explaining the flash-lag effect.

Fig 3 summarizes the main results when simulating the FLE with the dMBP model for *τ* = 100 ms, a realistic delay for human motion perception. In Fig 3-A, we plot the estimated positions of both static (flashing) and moving stimuli. The position of the moving dot shows a spatial lead similar to what was reported in the psychophysical literature. Fig 3-B illustrates the responses of the dMBP model together with a control condition, the position-based prediction (PBP) model where the predictive term relative to motion was discarded. This PBP model is simply defined by using the same equations but assuming that, first, the precision in Eq 19 is zero, meaning that the diffusion parameter *D*_*V*_ is infinite and second, that the speed prior defines an isotropic diffusion factor similar to that of the diffusion in position. Thus the PBP and dMBP models differ in regards of the information used (position only vs position and velocity) and thus of the shape of the diffusion (isotropic vs anisotropic). For the two models, we analyzed their estimated responses as spatial histograms with 50 bins over the range of horizontal spatial positions (that is, (−1, 1)). In particular, the positional bias reported for the FLE was computed as the inferred position of the moving dot at the instant at which the flashed stimulus reached its maximum precision, that is to say when the standard deviation of its inferred position was minimal. In Fig 3-B, we plot the two frames before and after that instant. As the flash was an unexpected event, this maximum was achieved after the fixed delay period and a variable processing delay that we robustly observed to be of about one frame (≈10 ms) in our simulations. It is evident from Fig 3-B that, at the moment at which the flash was maximally activating the dMBP model, the estimation of the position of the moving stimuli was ahead of the location of the flash. By comparison, the PBP model did not show this effect, suggesting that motion-based prediction is indeed necessary to account for the Flash-lag effect.

As expected, the speed of the moving dot affected the perceived spatial lead. When running the dMBP model with *τ* = 100 ms, the spatial lead of the moving dot monotonically increased with dot speed, until it reached a saturation value for very high speeds (Fig 3-C). This result is consistent with the empirical observations of Wojtach et al. [36] but also that reviewed in [47]. However, such nonlinear relationship between spatial lead and stimulus velocity was obtained without the need for a specific speed-tuned prior, as postulated in the model of Wojtach et al. [36]. The saturation observed for high speeds was concomitant with a sharp increase in the variance of the position estimates. Thus, inferred motion was less precise for higher speeds, despite the fact that (spatial) trajectory length remained constant. Again, this result is consistent with psychophysical data on global and local (dot) motion perception (e.g. [48–50]). Overall, the non-linear relationship between spatial lead and the dot motion speed results from both the optimal integration of information within the system and the decrease in precision of the sensory information at higher speeds. Thus, the dMBP model highlights the importance of having a probabilistic representation of visual motion in order to elaborate mechanisms which are able to compensate for neural delays.

### Standard FLE versus half-cycle FLEs

Next, we simulated the spatial lead of a moving dot in the case of half-cycle FLEs where the flash appeared either at the beginning or at the end of the motion trajectory. As discussed above, the half-cycle FLEs described by Nijhawan [28] have challenged the diagonal model of FLE. Therefore, our goal was to test whether our diagonal motion-based prediction (dMBP) can account for these different conditions.

**Fig 4:**
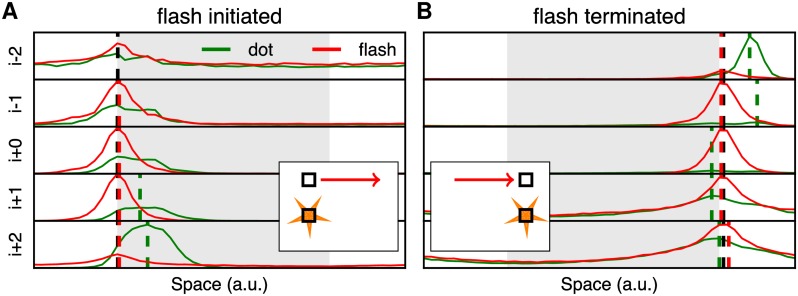
Both flash-initiated and flash-terminated conditions can be explained by the diagonal motion-based prediction (dMBP) model. With the same format as Fig 3-B, we plot the temporal evolution of the probability distributions of the inferred position for both the flashed (in red) and moving (in green) dots, in the (A) flash-initiated and (B) flash-terminated conditions. As in Fig 3-B, each curve corresponds to the five frames (respectively numbered from *i* − 2 to *i* + 2) centered on the time of the model’s maximal response to the flash. Dashed vertical lines indicate at each frame the estimated positions from the maximum a posteriori of the probability distributions for either the flash (red) or the moving (green) dot, together with the veridical position of the flashed dot (black). As expected, one can observe that the distribution of inferred positions is approximately correct for the flashed stimulus in all conditions. In the flash-initiated FLE condition, the distribution for the moving dot is biased towards its direction and develops very rapidly. Notice however that these biases are smaller than observed with the standard FLE. In the flash-terminated conditions, the bias is observed in the last frames before the maximum of the flash and then competes with another estimate with no bias which dominates near the moment of the flash’s maximum. Note that the a posteriori probability distributions around the flash’s maximum are very broad and indicate a high spatial uncertainty. Altogether, the absence of bias in the flash-terminated condition is similar to that reported psychophysically with human observers [28].

Fig 4 illustrates the model’s output for the two different half-cycle FLEs by plotting the probability distributions for the inferred positions of both the flashed (red curves) and moving (green curves) dots. The estimated perceived positions were computed as the maximum a posteriori values, across the five frames duration of the flash (respectively numbered from *i* − 2 to *i* + 2). These a posteriori probability distribution functions represent the peak (most probable) inferred position as well as the spatial uncertainty which are essential components in motion-induced perceptual shifts [38]. In the flash-initiated half cycle, the flash appeared at the beginning of the moving dot trajectory. The simulations unveiled two phenomena. First, the precision of the estimated positions of the flash gradually increased over time (lower left panel). Moreover, we observed that the center of the distribution of the inferred positions was always aligned with respect to the physical location of the flashing dot (see Fig 4-A). Second, ones can see a rapid sharpening for the position of the moving dot over time. This increase in precision was concomitant with a smooth shift of the moving dot’s perceived position along the motion direction, corresponding to the classical FLE. As such, our model simulate a position bias in the flash-initiated condition that is consistent with the psychophysical observations [28].

The dynamics was different in the flash-terminated condition (Fig 4-B). Consistent with the standard FLE, we observed first a bias in the two frames occurring before the maximum of the flash. Moreover, the distribution was very broad, consistent with a high uncertainty about the position of the dot. Gradually, the maximum of the position’s distribution shifts towards the flash location. Hence, near the moment of the flash’s maximum response, we obtained a bimodal distribution corresponding to a competition of the early extrapolated position with a second, unbiased distribution. At the time the flash was perceived, the two peaks corresponding to the estimated positions of both flashed and moving stimuli were now closely matched. These reported positions were now consistent with the classical psychophysical observations that, in flash-terminated cycles, there is no perceived spatial lead at the moment the moving dot disappears. When interpreting the disappearance of bias in the flash-terminated condition, Eagleman and Sejnowski proposed that the movement occurring before the flash was not sufficient to induce a flash-lag illusion and therefore proposed an alternative theoretical framework, the postdiction model [27]. Other studies have reported that under some stimulus conditions, the FLE does occur in the flash-terminated cycle (e.g. [51, 38]) in particular when the uncertainty about the position in space of the moving stimulus is high. The simulated dynamics of the estimated position during the flash-terminated cycle shows that the probabilistic representation of visual inputs underlying the dMBP model is sufficient to reconcile those apparently contradictory results. Thus, the dMBP provides a powerful framework to account for the different variants of the FLE and a broad range of their experimental conditions. We will now explain why the dMBP model can account for these different variants of the FLE.

**Fig 5:**
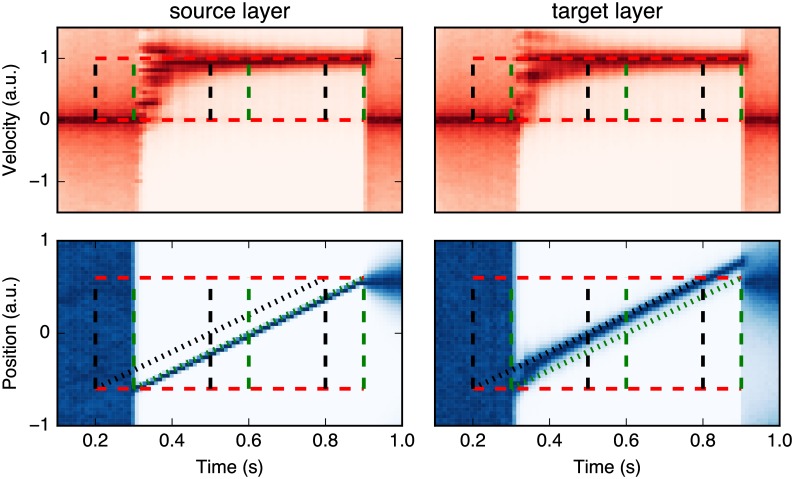
Histogram of the estimated positions as a function of time for the dMBP model. Histograms of the inferred horizontal positions (blueish bottom panel) and horizontal velocity (reddish top panel), as a function of time frame, from the dMBP model. Darker levels correspond to higher probabilities, while a light color corresponds to an unlikely estimation. We highlight three successive epochs along the trajectory, corresponding to the flash initiated, standard (mid-point) and flash terminated cycles. The timing of the flashes are respectively indicated by the dashed vertical lines. In dark, the physical time and in green the delayed input knowing *τ* = 100 ms. Histograms are plotted at two different levels of our model in the push mode. The left-hand column illustrates the source layer that corresponds to the integration of delayed sensory information, including the prior on motion. The right-hand illustrates the target layer corresponding to the same information but after the occurrence of some motion extrapolation compensating for the known neural delay *τ*.

A first step was to further detail the internal dynamics of the dMBP model during the motion of the dot. As shown in Fig 5, we investigated the temporal dynamics of the estimations of both position and velocity by plotting their spatial histograms as a function of time, over the complete trajectory. We focused on three different epochs of motion trajectory, corresponding to the standard, flash-initiated and flash-terminated conditions. For each epoch, the vertical dotted black lines indicate the physical time of the flash and the green lines signal the delayed input with the known delay (*τ* = 100 ms). Both the source and target layers are illustrated for each of three different phases. First, we found a rapid build-up of the precision of the target after the first appearance of the moving dot (at *t* = 300 ms). Consistent with the Frölich effect, the beginning of the trajectory was seen ahead of its physical position as indicated by the maximum of probability distributions lying in between the oblique green and back dotted lines. During the second phase, the moving dot was correctly tracked as both its velocity and position are correctly inferred. In the source layer, there was no extrapolation and the trajectory followed the delayed trajectory of the dot (green dotted line). In the target layer, motion extrapolation correctly predicted the position at the present time and the position followed the actual physical position of the dot (black dotted line). Finally, the third phase corresponded to the motion termination. The moving dot disappeared and the corresponding activity gradually vanished in the source layer at *t* = 900 ms. However, between *t* = 800 ms and *t* = 900 ms, the dot position was extrapolated and predicted ahead of the terminal position in the target layer. At *t* = 900 ms, sensory visual motion information was now absent and the prior for slow speeds dominated. Both the quick drop of the estimated velocity after the dot’s disappearance and the diffusion of this information in both position and velocity spaces led to the progressive extinction of position extrapolation ahead of the sensed position. Consistently with this new information, the position information was now gradually extrapolated thanks to the broad, zero-centered prior distribution for speeds. As such, the inferred position in the target layer was now extrapolated isotropically from that of the source layer at *t* = 900 ms, that is to say, at the terminal horizontal position. Although this distribution was much less precise, the average position of the moving dot at flash termination was invariably perceived at the same position as that of the flash.

The termination epoch illustrates some key differences between our probabilistic model and previous theoretical explanations. The dMBP model explicitly differentiates between a zero motion (i.e. “I know that the stimulus does not move”) and an absence of knowledge, as represented by the prior distributions for velocities. In particular, we do not need to postulate the existence of a resetting mechanism. For instance, the FLE is present at termination when introducing spatial uncertainty (for instance at a higher eccentricity) but disappears again in the same configuration when the dot stops and does not not disappear [38]. A second key difference is the introduction of a compensation for the latency which is controlled by the precision of the sensory and internal beliefs. The important distinction is that the system is tuned to give the most probable state at the current time even if the incoming information is from the past (delayed by *τ*). In particular, at the end of the trajectory, the system updates its prediction after the delay of *τ* = 100 ms, according to the collected information at *t* = 800 ms and which is sensed at *t* = 900 ms. At this particular moment, instead of keeping the prediction that the dot moved during this period, the dMBP model updates its state with the knowledge that the dot disappeared at the physical time corresponding to motion offset and is more likely to be near the last observed position at *t* = 900 ms (and that corresponds to the physical time *t* = 800 ms). One prediction of the dMBP model is therefore that, with a long enough delay (as is the case here), the predicted position should be first estimated ahead of the actual position and then, *with hindsight*, shifts back to the position accounting for the end of motion (see Fig 4-B). Overall, this dynamics explains the perceptual difference observed between the flash-terminated and flash-initiated FLEs and provides a simple and parsimonious alternative to the postdiction theory.

### The FLE and beyond: Motion reversal

**Fig 6:**
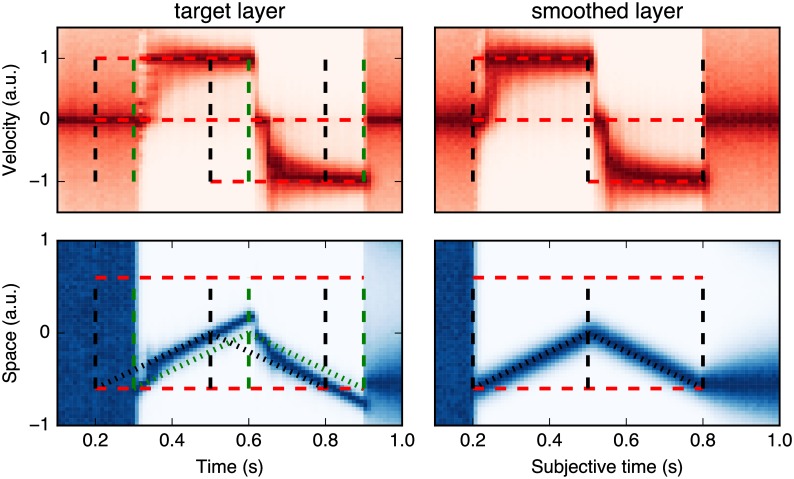
Estimating the dot position from the dMBP model during the motion reversal experiment. In the motion reversal experiment, the moving dot reverses its direction at the middle of the trajectory (i.e., at *t* = 500 ms, as indicated by the mid-point vertical dashed line). In the left column (target layer) and as in Fig 5, we show the histogram of inferred positions during the dot motion and a trace of its position with the highest probability as a function of time. As expected, results are identical to Fig 5 in the first half period. At the moment of the motion reversal, the model output is consistent with previous psychophysical reports. First, the estimated position follows the extrapolated trajectory until the (delayed) sensory information about the motion reversal reaches the system (at *t* = 600 ms, green vertical dashed line). Then, the velocity is quickly reset and converges to the new (reversed) motion such that the estimated position “jumps” to a position corresponding to the updated velocity. In the right column (smoothed layer), we show the results of the same data after a smoothing operation of *τ*_*s*_ = 100 ms in subjective time. This different read-out from the inferred positions corresponds to the behavioral results obtained in some experiments, such as that from Whitney and Murakami [23].

Extending the previous results, we investigated the inferred position when the motion of the dot is not interrupted, but reversed. As such, we simulated the experiment reported by Whitney and Murakami [23] and modeled by Rao et al. [45]. In this variant of the FLE, the moving dot reverses direction at the middle of the trajectory, and then maintains its new trajectory. To implement this stimulus, we used the same stimulus as in Fig 3, but mirrored vertically the image for the frames occurring in the second half of the movie. Results are shown in the left column of Fig 6, using the same format as the target layer in Fig 5. As expected, the model’s behavior is consistent with that observed with the flash-initiated cycle condition. First, the estimated position follows the first half of the trajectory and continues to be extrapolated after the time *t* of reversal and until the moment *t* + *τ* at which the sensory evidences about the motion reversal has reached the system. From this moment in time, the source layer updates the target layer according to the new visual information. As in the previous case with the flash-terminated FLE, the estimated velocity is rapidly updated and converges to the new motion direction. Using the parodiction hypothesis, the model updates at this instant the velocity used in the extrapolation of the present position as it acquires this novel knowledge (that is, with some hindsight). The estimated position in the target layer thus “jumps” to the new location and then follows the second half of the trajectory.

Overall, the model’s behavior is qualitatively similar to the filtering model reported by Rao et al. [45]. Under some generic hypothesis about the noise distribution, the dMBP model is in fact equivalent to a Kalman filter with a fixed delay [8]. It is therefore consistent with the optimal filtering model of Rao et al. [45]. At first sight, the response of our model may seem at odd with the behavioral results reported by Whitney and Murakami [23] where no overshoot (and thus no jump) was found in the estimated position after motion reversal, as predicted in the optimal smoother model proposed by [45]. This model extrapolates the current position knowing the past positions within a temporal window corresponding to some subjective latency. It is based on the postdiction hypothesis postulating that the position that is accessible to visual awareness is evaluated after some additional delay *τ*_*s*_. Thus, position is reported in the reference frame of a delayed, subjective time which is the same for the two stimuli (the moving and flashed dots). Similarly to that model, we may use our probabilistic framework to update the information after this delay: The postdiction hypothesis thus states that the evaluation for the position at the present time *t* is done in the future at time *t* + *τ*_*s*_. The information at this time is *p*(*z*_*t*+*τ*_*s*__|*I*_0:*t*−*τ*+*τ*_*s*__) and similarly to the extrapolation which is performed over future times (see Eq 5), we may extrapolate over past times (that is, backwards in time) using a similar hypothesis:

$$p\left(z_{t}|I_{0:t-\tau +\tau_{s}}\right)=\int dz_{t}\cdot p\left(z_{t}|z_{t+\tau_{s}}\right)\cdot p\left(z_{t+\tau_{s}}|I_{0:t-\tau +\tau_{s}}\right)$$ (20)

The output of this transformation is shown in the right column (labelled smoothed layer) of Fig 6, with *τ*_*s*_ = 100 ms and the values of subjective time being realigned to physical time for the sake of clarity. Now, our results show qualitatively no overshoot and the model’s dynamics is similar to the optimal smoother model proposed by Rao et al. [45]. Note that a similar transformation applied to the flash-terminated cycle would qualitatively smooth the estimate of the position using past frames and thus enhance the absence of bias in this case.

This transformation applied to the probabilistic information illustrates two key properties of the dMBP model. First, it shows that the model can explain the experimental data from Whitney and Murakami [23] by explicitly modeling the decoding used in this experiment. In particular, our model can explain why the spatial position of the moving dot begins to deviate *before* the random time of the reversal. Such probabilistic framework can also account for the contradicting results obtained in the flash-terminated cycle where, for instance the FLE was reported by Eagleman and Sejnowski [27] but not by others [38, 51] and was found to be in fact dependent upon the uncertainty about the position of the moving dot. Second, it demonstrates the flexibility of the representation used in the parodiction hypothesis and its capacity of subsuming the differential latency, motion extrapolation and postdiction hypotheses. Hence, the diversity of alternative models drawn to account for the various FLE experiments is not necessary, thanks to the probabilistic mechanisms used by the visual system to decode this information.

### Modeling the effects of stimulus contrast and duration on the flash-lag effect

We have shown above that the dMBP model can explain the different variants of the FLE. In order to further understand how the precision of the probabilistic representation shapes the FLE, we manipulated a small set of key visual parameters which are known to tune the dynamics of the dMBP model: stimulus contrast and duration. As shown in our previous modeling study about the role of motion extrapolation in object tracking [17], decreasing the contrast of a moving stimulus results in a modified dynamics of the predicted state. One important consequence is that a predictable, moving stimulus may then be detected at lower contrasts than an unpredictable flash since the system integrates sensory information along the motion trajectory. This is consistent with previous psychophysical observations about single dot motion detection in a noisy display [52]. However, since contrast differentially modulates the processing of either flashed or moving stimuli, it is important to investigate its effects upon simulated FLE. A prediction is that a flashed stimulus shall be more affected by lowering the contrast than a moving one, resulting in different effects upon FLE. We could then unify several empirical evidences that have led to different theoretical interpretation of FLE (e.g. [33, 38]).

**Fig 7:**
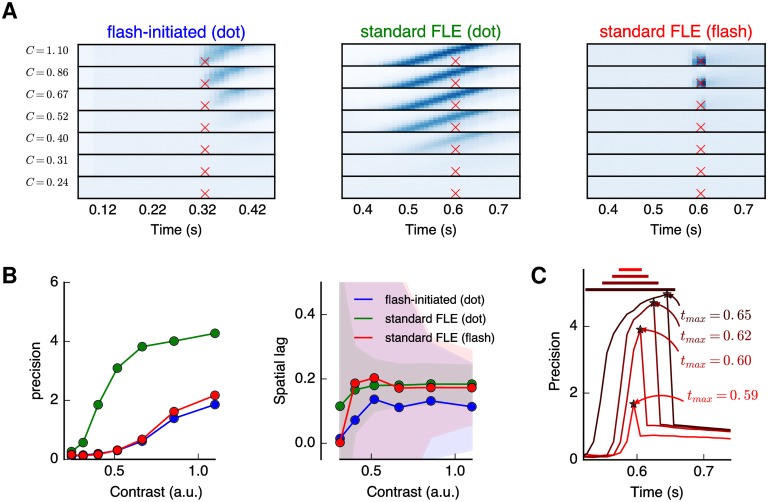
Dependence of the FLE with respect to contrast and duration of the stimuli. (A) Using the same data format as in Fig 5, we show the spatial distribution of the estimated response (zoomed around its physical position at the perceived time of the flash at full contrast which is indicated by a cross) for different different relative contrast levels *C* indicated at each row. The different columns correspond from left to right to different conditions where the contrast of the dot is manipulated (first two columns)—respectively at the beginning of the cycle (i.e. flash-initiated) cycle, the mid-point (i.e. standard cycle)— or where the contrast of the flash is varied (right-end column). Note that in the standard FLE case (middle column), the model already responds to very low values of dot contrast in a nearly all-or-none fashion. By comparison, the responses to the dot or the flash during the initial phase of the trajectory gradually increased with contrast. In particular, the dot’s lag seems to increase more rapidly with respect to contrast. (B) These qualitative results are best illustrated by plotting in the first column the precision of the response as measured by the inverse standard deviation of the estimated position as a function of contrast of the different conditions. Coherent with the results illustrated in (A), the precision of the representation varies gradually against contrast of the flash or moving dot in the early phase whereas it changes more rapidly and abruptly as a function of the moving dot’s contrast in the standard FLE. Consequently, we estimated in the second column the spatial lag that is expected when changing the contrast of the stimuli (± one standard deviation). Coherently with psychophysical results, increasing the contrast of the moving dot gradually increases the FLE in the flash-initiated cycle but has only limited effects in the standard FLE when above a given precision as it rapidly reaches a saturating value of ≈0.2 corresponding to a full compensation of the fixed delay. Consistent with [38], these results show the role of spatial uncertainty in dynamically tuning the estimated position and, ultimately, in influencing the spatial lag in the FLE. (C) As shown by [47], flash duration modulates FLE. We show here the precision for the flash as a function of time with respect to duration. While the peak remained at *t* = .5 s (that is, at *t* = .6 s when including the delay), we tested for different durations, respectively .03, .05, .08, .13, .25 in s (as marked by colored horizontal bars). The respective measured time to reach the maximal precision are given by *t*_*max*_ (in s), showing that precision was high for *T* ≥.05 s (that is, 50 ms). Notice that this value was used for all the experiments described above.

We first simulated the relationship between FLE and the relative contrast between the dot and the flash, in two different conditions, the standard cycle (i.e. flash at mid-point) and the flash-initiated cycle. These two conditions correspond to cases where we observe the precision of the dot position early or late along a similar motion trajectory. The results are illustrated in Fig 7-A. Using the same conventions as in Fig 5, the estimated distributions of horizontal positions are plotted against time. These spatial distributions are the average over 20 trials and are shown for a time lapse centered around the maximal precision of flash stimulus. The first two columns show these distribution as estimated at two different time epochs, that is early or late during a motion trajectory, respectively corresponding to flash-initiated or standard FLE conditions. Early distributions are strongly sensitive to dot and flash contrast, with almost no reliable estimate at very low contrast (i.e. below *C* ≈ 0.4). By comparison, the distributions for the dot at the standard FLE cycle emerge at very low contrast, and rapidly reach their stable solution. In the temporal domain, we also observed that for lower contrasts, the dynamics of integration became slower such that the peak was reached slightly later. Fig 7-B quantitatively reports the effects of contrast on the precision of the estimated position (that we define here as the inverse of the standard deviation). During the flash-initiated cycle, we found a smooth increase in precision with higher contrast of either the moving dot (blue curve) or the flash (red curve). The contrast-precision function was much more step-wise in the standard cycle condition where dot position is estimated at the mid-point of the motion path. The precision was also much larger in this later condition. From these, we estimated the spatial lag (second column, Fig 7-B) against contrast. A prediction of our model is that a broader distribution in position, such as observed with a lower stimulus contrast, would be associated with a coarser velocity estimation. Such uncertainty would impact the extrapolation of positional error and thus the spatial lag. As expected, we found a large increase in the spatial lag as the contrast of the dot increased in the flash-initiated cycle (blue line). This effect was largely reduced in the classical FLE condition (green line). We also varied the contrast of the flash and found, for a contrast above *C* ≈ 0.5, a decrease in the spatial lag as contrast increased, as expected from the precision measurements. Notice that estimating the spatial lag for very low contrasts is very unreliable given the high spatial uncertainty in the position estimation (see Fig 7-A, lower rows).

Overall, the dMBP model can simulate the main effects of the relative contrast between the flash and the dot, in different conditions. These simulated relationships can be compared with previous empirical studies that investigated the impact of contrast upon perceived FLE [33, 38] (see [29] for a review). Consistent with previous results [16], we observed that contrast affects the precision of position estimates mostly at the beginning of the motion trajectory, as in the flash-initiated cycle for instance. Since the model started to efficiently track the dot at a later time point, a weaker flash-initiated FLE is observed at lower contrast values. By comparison, at trajectory points that are more distant from the motion onset, as in the standard FLE condition, the tracking remained very precise at low contrast and thus, the size of the FLE was only marginally affected. Overall, our model unifies the different observations that the uncertainty generated by a lower effective contrast (and other stimulus conditions) results in lower FLEs [38]. Our model also allows to compare the relative effects of the moving versus flashed dots. For instance, the spatial lag increases with the relative contrast of the moving dot, but slightly reduces with it. This is consistent with a previous empirical study [33]. It shall be noticed that these authors have manipulated the relative luminance of the stimuli rather than contrast. However, assuming a stationary exogenous noise source, luminance and contrast changes shall yield to similar model outputs. Thus, our model is compatible with their results but it does so by compensating for a fixed delay, without the need of postulating different latencies for moving and flashed inputs [33]

As a final control, we manipulated flash duration. While we did not observe any effect of this stimulus parameter on the mean estimated position, the precision was dynamically modulated by it (see Fig 7-C). We observed a characteristic integration time controlled by the *σ*_*I*_ parameter of the likelihood model (see Table 1). By consequence, precision at the peak decreased with very short durations (3 frames, that is, 30 ms). Then, the precision quickly stabilized over the stimulation duration, consistently with the stationary solution of the master equations of the MBP model. The varying timing corresponding to the optimal precision with respect to flash duration thus has a consequence on the FLE. However, such consequences are harder to estimate in our framework, in particular for durations longer than *τ*. In these particular cases, the maximum of the estimated position was ambiguously defined and did not necessarily correspond to the middle of the flash. Rather, since information was progressively integrated, the precision was in theory increasing and should be reported to peak as the flash disappears. As such, the spatial lag in the FLE should decrease with longer flash durations. This is consistent with experimental reports on the disappearance of the FLE for flash durations longer than 80 ms [47]. This result also justifies that in the present study we used a short flash of 5 frames, that is, 50 ms.

## Discussion

### Motion-based prediction, position coding and the flash-lag effect

In this computational study, we have investigated the role of prediction in a challenging and popular example of motion-induced position shifts, the flash-lag effect (FLE). When elaborating a novel theoretical framework for both experimental and modeling studies on FLE is an essential step to better understand the perceptual mechanisms of visual position coding and the compensation for the delays inherent to any neural mechanism. As stated above, at least three main explanations of the FLE have been proposed in the literature but none of them can account for all the properties of the FLE. Based on our previous work on predictive coding and visual motion processing [16, 17, 53, 44], we propose that the FLE and its different variants are a mere consequence of a motion-based predictive coding mechanism that is compatible with low-level visual processing.

Our simulations demonstrate that the motion of a localized target can shift the estimation of its position along the motion direction. We introduced a second flashed input lasting 5% of the trajectory length which we presented at the beginning, middle and end of the trajectory, matching the three main empirical cases used to probe the FLE [28]. We show that the diagonal motion-based prediction (dMBP) model can explain the presence or absence of a spatial lead of the moving stimulus in all these three variants of the FLE. In particular, the model can simulate one main perceptual fact: the spatial offset is seen in the flash initiated cycle but not in the flash terminated cycle. For the first time, our model can thus provide an explanation for all three empirical results, as a consequence of the optimal extrapolation in the positional coding of a moving object in the direction of its motion.

This approach can be seen as an extension of the motion extrapolation theory originally proposed by Nijhawan [4] and which has been highly disputed over the last two decades. Our dMBP model is a generic motion estimation algorithm which uses a probabilistic representations that explicitly estimates the distribution of beliefs about the motion of the stimulus by using both position and velocity information about the object trajectory. The dMBP model is able to compensate for a known neural delay and to differentiate between moving (predictable) and static, flashed (unpredictable) objects. Moreover, we have shown that the precision of this belief tunes the gain of the predictive coding mechanism similarly to the range of psycho-physical observations. It thus provides a rationale for coding the estimated position of both the flashed and the moving stimuli in the FLE.

### Experimental evidences for the dMBP model

This study provides several theoretical insights on the role of motion signals in the dynamical representation of objects’ positions and its dependency upon several visual factors such as speed, contrast, duration or the timing of the flash respective to the motion trajectory. Several psychophysical and neurophysiological studies have shown that the perceived position of a moving object is shifted into the direction of motion, a phenomenon called motion extrapolation (e.g. [4, 14, 54, 15, 55–57]). Motion extrapolation has been assumed to be caused by an anisotropic pattern of connectivity between position-coding cells in the retina [4, 14, 54] or the early visual cortex [15]. Thus, different modeling studies have simulated motion-induced position shifts by using position-tuned cells and dynamical effects of lateral interactions in neural networks [58–61, 11]. We show here a similar behavior, resulting from the interactions between position and velocity coding. We suggest that a predictive bias of the neural representation of position can result from these interactions, a general rule of early visual processing stages within retinotopic maps. Moreover, contrary to most of previous models, the dMBP model can simulate the relationships between the perceived positional shift in the FLE and properties of the object’s motion. This later relationship is coherent with previously reported psychophysical studies [36] and highlights the interactions between position and velocity coding.

In particular, from previous computational studies in motion extrapolation, one may expect a smaller spatial lead in the flash-initiated cycle. In the earliest frames of the trajectory, position estimation will in fact lag behind the actual, physical position of the stimulus. We have not found this effect with the dMBP model. Only a few frames within the flash duration were sufficient to modulate the position estimation of the moving object and to compensate for the delay. Thus, the dMBP model does predict no significant difference between the size of spatial leads simulated in flash-initiated and standard FLEs. On the other hand, in the response of the dMBP model to the flash terminated FLE, there is a close match between the estimated positions of both flashed and moving stimuli, consistent with the psychophysical evidence. This effect is easily explained by the interplay between the input sensory layer and the predictive target layer at the instant when the flash is sensed. At this precise time, the estimated velocity for the moving dot is updated with hindsight to a distribution centered on a zero speed, causing a static extrapolation at the end of the trajectory and correcting for the wrongly assumed motion of the dot during the delay period. This causes the “jump” from the extrapolated trajectory to that observed at the instant of the flash. Such prediction should be investigated at perceptual and physiological levels in future work.

Another empirical aspect of the FLE is the dependence of the spatial lead with respect to contrast [33, 38]. In fact, the dMBP model is based on the accumulation of information along the trajectory, without any pre-determined contrast gain control mechanism. As a result, a low contrast would only result in a slower build-up of the tracking behavior, as previously observed [16]. Here, the dMBP model highlights two important points in the contrast dependence of the FLE. First, for the flash initiated cycle, a higher contrast would result in a larger spatial lead of the moving object. Second, for both standard and flash terminated cycles, that is, for positions located late enough along the object’s motion trajectory, the dependence of spatial lead on contrast can be mostly explained by the contrast of the flashed input, as shown by the relationship illustrated in Fig 7. Indeed, consistent with experimental evidence [38], the model’s response to a low contrast flashed stimulus takes longer to reach the detection threshold such that the moving stimulus will further advance along the trajectory before the input arrives, resulting in a larger positional lead.

### Shortcomings of the motion extrapolation theory

It is important to highlight some key differences between our dMBP model and previously published models based on motion extrapolation. In the literature, two main shortcomings of motion extrapolation were raised. First, it was found experimentally that the spatial lead of moving object in flash-initiated cycle is comparable with the spatial lead observed in standard FLE. This is in contradiction with the fact that, according to the motion extrapolation hypothesis, the positions of the two objects should not be distinguishable enough during the earliest phase of the motion trajectory. By contrast, the dMBP model shows a quantitatively similar positional lead in flash-initiated cycle. This result resolves an apparent shortcoming of the motion extrapolation theory: the duration of the flash is enough to initiate the integration of motion signals along its trajectory and to correctly compensate for the positional error caused by neural delays.

Another classical shortcoming of the motion extrapolation theory is related to the flash-terminated cycle. Empirically, no spatial lead or overshoot is perceived for flashes occurring at the end of trajectory whereas the motion extrapolation model would predict one. Because of this contradiction, several of the existing models decided to ignore the flash-termination FLE. For instance, the model of Wojtach et al. [36] did not use probabilities to represent motion but simply the optimal estimate of speed using an empirical prior. One consequence is that, as in the motion extrapolation model of Nijhawan [28], such models do not predict the lack of effect for flash terminated cycle. In contrast, the dMBP presented herein implements a velocity-dependent position extrapolation based on a probabilistic representation, which is flexible enough to cease prediction of the trajectory after the motion’s stop.

As argued by Nijhawan [28], the motion extrapolation model is a powerful hypothesis for the estimation of the motion of smoothly moving objects. However, as soon as a significant transient input modifies the stimulus, other supplementary mechanisms must be postulated to modulate the extrapolation computation. In the dMBP model, the internal model of visual motion progressively integrates confidence about the trajectory. We have shown previously that this mechanism may be sufficient to fill a short blank along the trajectory [17]. Still, even in this “tracking state” [16], the dynamical system is sensitive enough to be modulated by changes of the stimulus’ state, such as a sudden stop or a motion reversal. Knowing the neural delay, this stop will correct the path of the trajectory over this period in order to update the actual position of the object at the present time. A strong improvement over other motion extrapolation models is that the dMBP model does not have to postulate any other specific mechanism such as the resetting mechanism in the postdiction hypothesis. This correction is entirely handled within the motion-based predictive mechanism by the probabilistic computations. We thus provide a new computational evidence that motion extrapolation is a successful explanation for the FLE when separating the smooth prediction of the trajectory which is based on motion coherency (i.e. *p*(*z*_*t*−*τ*_|*I*_0:*t*−*τ*_)) from its projection at the present time (i.e., *p*(*z*_*t*_|*I*_0:*t*−*τ*_)). Therefore, the inferred trajectory does not necessarily need to be smooth as it can be corrected with the hindsight corresponding to the known delay. In summary, our new theoretical framework of the motion extrapolation hypothesis can reproduce the experimental data of the main variants of the FLE, a clear advantage over all other previous models. Overall, we show that an internal representation of object motion can provide a substrate for the coherency of its perceived motion despite the existing neural delays as well as a transient interruption in the sensory inflow [16, 17]. It can thus provide a more reliable interpretation of the visual input. Herein, we have investigated the functional advantages of using both position and velocity information in building this internal model.

### Comparison with other neuromimetic models for FLE

How does our theoretical work relate to previous modeling work using neuromimetic networks? The neural field model of Erlhagen [58] is the most relevant study demonstrating that the emergence of a spatial lead in the FLE can result from the interplay between an internal model and a feedforward flow of stimulus-induced neural activity (but see also [11]). Their network was made of excitatory and inhibitory populations of position coding cells. Motion extrapolation of the trajectory emerges from both lateral interactions and network’s dynamics. This model showed that the priming of a position field is caused by the accumulation of sub-threshold activities of excitatory populations. Our model also highlights the role of the internal model of trajectory, where the internal model was based on the accumulation of motion information. The main difference between the dMBP and Erlhagen’s models is the use of velocity information and not only position coding. Still, both models stress the critical role of sub-threshold neural activities in the emergence of motion anticipation and extrapolation. In Erlhagen’s model, this is achieved by finely setting some parameters of the neural field. On the contrary, motion anticipation and extrapolation both emerge in the dMBP model from the probabilistic accumulation of motion-based position estimation along the motion trajectory.

Only a few other neural network models have directly addressed both delay compensation, motion extrapolation and FLE. For instance, the model of Baldo and Caticha [59] investigated how motion extrapolation and FLE may arise from a simple feed-forward network of leaky integrate and fire neurons. Other neural network models have addressed the question of neural delays and motion extrapolation at the single neuron level [60, 61]. In these models, neurons are sensitive to the rate of change in the input and, via extrapolative activations they can estimate the state of external world at time *t* + *τ* instead of time *t*. On one hand, facilitatory activity is derived from the present and past activity of network and on the other hand, synaptic efficacy implements a smoothness constraint in the spiking activity which implements motion coherency. Therefore, spiking activity is extrapolated in the direction of change via a spike-time dependent learning rule along with facilitated synapses. However, these models have investigated spatial priming of neurons via lateral and facilitatory connections, ignoring the facilitatory effect that may arise from velocity coding.

Last, the dMBP model is partly consistent with the theoretical framework underpinning the postdiction model of FLE [27]. Both models infer variables using a state space model from a dynamical model of the moving stimulus. Prediction and postdiction mechanisms can be related to two essential components of dynamical systems in engineering: filtering and smoothing, respectively. The former has access to the immediate past of the signal to estimate the current state and the later model estimates the current state based on the immediate future. The main difference between the two models is that we use a filtering approach while the formulation proposed by Rao et al. [45] rather adds a smoothing component. As we have illustrated in the motion reversal experiment, we can also easily integrate such a smoothing but we have shown that this additional component is not necessary to explain other aspects of the FLE. As such, both models can reproduce the Fröhlich effect as a mis-estimation of earliest part of the trajectory, but the postdiction model interprets that the visual system attributes a position to a visual event only when it accumulated enough sensory evidence within the sensory integration time window. However our simulations suggested the important role of velocity information in this compensation, while the postdiction theory would propose that the position of stimulus is always pushed forward based on trajectory information received in a short time interval after time *t*. Lastly, contrary to the dMBP model, the postdiction model fails to account that in the standard FLE, no velocity increment for moving stimulus has been reported psychophysically [28]. In brief, while having similar mathematical formulations, the postdiction and dMBP models make different predictions, in particular regarding the earliest part of the motion trajectory. These predictions could be tested psychophysically in future work.

### Parodiction: An unified model for motion extrapolation

Our computational study suggests that the psychophysical evidences for the different variants of the FLE are the signatures of a fundamental neural mechanism that attempts to represent as best as possible the available information at the veridical physical time. The central nervous system is a complex dynamical, distributed machinery for which timing is essential. However, there is no evidence for the existence of a central clock as used in modern computers. Instead, neural computations are thought to be based on an *asynchronous* processing machinery. Even worse, some of these information bits are retarded by a range of inevitable transmission and computing delays. We suggest that an explicit neural representation of variables’s trajectories could compensate for these delays in order to represent each physical input at its present, physical time. Knowing these delays, motion extrapolation pushes the estimated position forward using the trajectory’s information. This paper thus proposes that the visual system exhibits the trace of an universal neural signature of a predictive processes compensating for neural delays. As such, in contrast to prediction and postdiction, a better term to explain this neural signature is *parodiction*, from the ancient Greek *παρóν*, the present time.

In the dMBP model, the dynamics for position coding is based on optimizing the probabilistic accumulation of information along the trajectory such as to be the most consistent at the present time. The dMBP model builds motion estimations at time *t* based on sensory information from the past (at time *t* − *τ*) and thus explains how neural delays may be compensated in visual processing. As a consequence, our model should be considered as an effort in understanding why phenomena such as the FLE should emerge. An utterly important question remains as to *how* this is achieved. Such attempts were advanced in several studies using a single paradigm for the different variants of the FLE [11]. Further modeling accounts were proposed to show that such a function could be implemented using asymmetric traveling waves [62] (see [15] for a more formal account). Our model offers to bridge and unify these different theories beyond the existing debate between proponents of differential latency and motion extrapolation. In particular, we propose an architecture which is parameterized by an anisotropic pattern of connectivity (see Fig 2-C). Key in that endeavor is a more precise knowledge as to how neural activity can dynamically code or decode a probabilistic representation of motion.

More generally, our new parodiction theory anchors any neural representation to the actual present time. Indeed, when considering any neurophysiological recording it is often assumed that the exact timing of each event, such as a spike, is known by the corresponding neural system relative to an absolute clock (for instance, that of the experimenter). However, the situation is different when overturning the problem relative to each individual neuron: For a neuron, the absolute time is unknown and only some relative timing of other neural events (their origin being sensory, motor or associative) may be known from previous experience. Also, the neuron has no access to past information and can only use traces of past neural activity at the present time. We claim that this problem is a generic problem in neuroscience as it raises the problem of the neural representation in time [8]. To conclude, we have proposed a theory, parodiction, which accounts for the FLE, but whose predictions remain to be validated in neurophysiology.
